# Case report: Urothelial carcinoma of the renal pelvis with trophoblastic differentiation: A rare case report and review of literature

**DOI:** 10.3389/pore.2023.1610856

**Published:** 2023-02-10

**Authors:** Zhuo Wang, Jinsui Wang, Wenwen Zhang, Daoying Wang, Xiaojun Wang, Xiaoqin Liang

**Affiliations:** ^1^ Department of Pathology, Gansu Province People’s Hospital, Lanzhou, Gansu, China; ^2^ Department of Radiology, Gansu Province People’s Hospital, Lanzhou, Gansu, China; ^3^ Department of the Centre of Positron Emission Tomography-Computed Tomography, Gansu Province People’s Hospital, Lanzhou, Gansu, China; ^4^ Department of Respiratory Medicine, Gansu Province People’s Hospital, Lanzhou, Gansu, China

**Keywords:** urothelial carcinoma, renal pelvis, trophoblastic differentiation, beta-human chorionic gonadotropin, syncytiotrophoblastic cells

## Abstract

We report a rare case of urothelial carcinoma (UC) of the renal pelvis with trophoblastic differentiation that occurred in a 55-year-old male patient. The patient presented with gross hematuria and paroxysmal lumbago pain 5 months ago. The enhanced computed tomography (CT) scan demonstrated a large space occupying lesion in the left kidney and multiple retroperitoneal lymph node enlargements. Histologically, high-grade infiltrating urothelial carcinoma (HGUC) contained giant cells which were positive for beta-human chorionic gonadotropin (β-hCG). Three weeks after resection, positron emission tomography and computed tomography (PET-CT) scan showed multiple nodules of metastasis in the left renal region, extensive systemic muscle, bone, lymph node, liver and bilateral lung metastases. The patient underwent bladder perfusion chemotherapy and gemcitabine combined with cisplatin chemotherapy regimens. This is the eighth documented case of UC of the renal pelvis with trophoblastic differentiation. Due to its rarity and extremely poor prognosis, it is important to clarify the characteristics of the disease and make an accurate and prompt diagnosis.

## Introduction

Specific subtypes of urothelial carcinoma (UC) have been studied and reported to be associated with pathological features, such as pathological staging and grading, lymphovascular invasion (LVI) and tumor recurrence. Squamous and glandular differentiation are the most common histological subtypes of UC. Urothelial carcinoma with trophoblastic differentiation (UCTD) is a rare and specific subtype of high-grade infiltrating urothelial carcinoma (HGUC) with syncytiotrophoblastic cells and positive immunohistochemical (IHC) staining for β-hCG. However, to our knowledge, most reported cases are from the urinary bladder, with only seven original reports concerning this specific subtype of renal pelvis UC reported in English literature (1–7). Herein, we report another case of a 55-year-old man with UCTD involving the renal pelvis. In order to reveal the characteristics of UCTD, we summarize the clinicopathological characteristics, surgical treatment, and prognosis in the review.

## Case report

**FIGURE 1:**
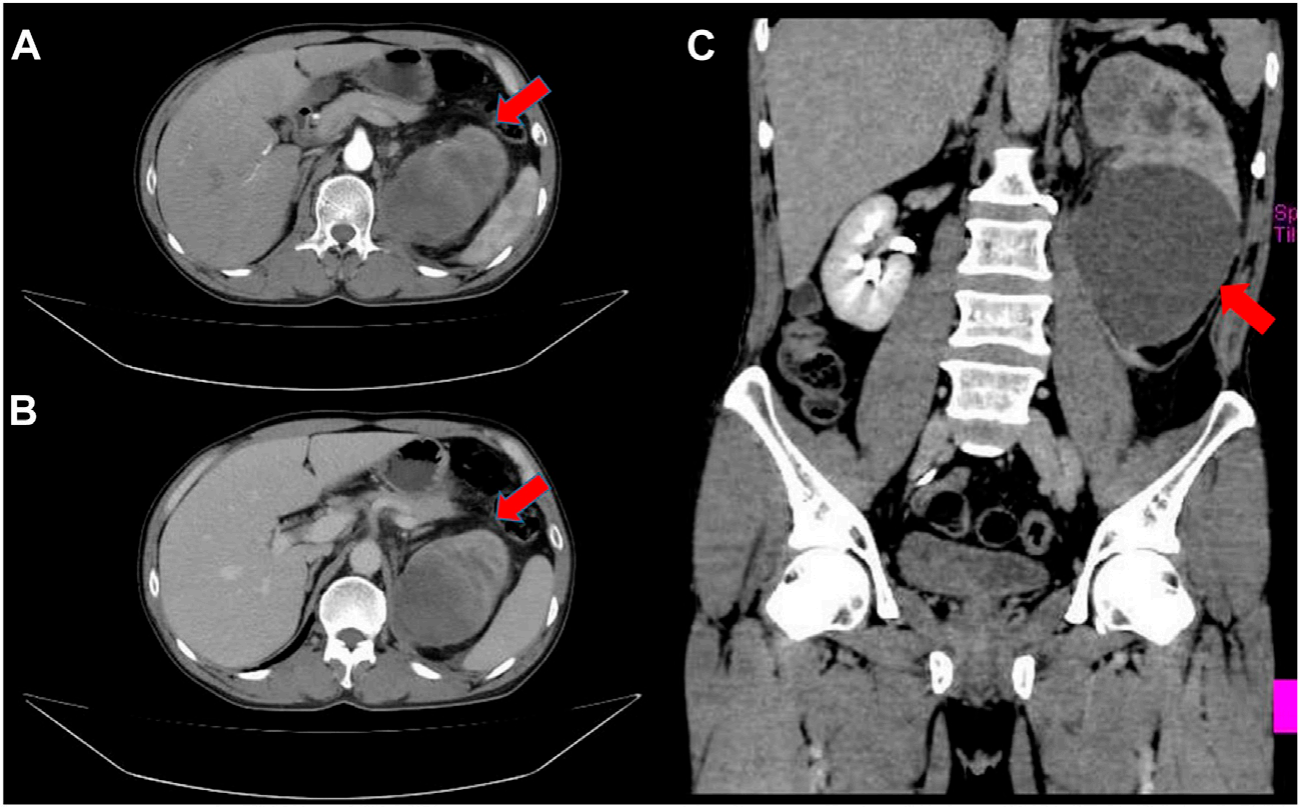
Enhanced Computed tomography revealed a large cystic solid tumor in the left kidney. **(A)** An arterial enhancement (red arrow); **(B)** A venous enhancement (red arrow); **(C)** Coronal plane (red arrow).

A 55-year-old male patient presented with gross hematuria and paroxysmal lumbago pain 5 months ago. Smoking history and family history of tumor was denied. The enhanced computed tomography (CT) scan revealed a 14.3 × 9.4 × 6.8 cm cystic solid mass of the left kidney with retroperitoneal lymph node enlargement (Figures 1A–C). Serum levels of β-hCG were not examined before operation. Then the patient underwent radical nephrectomy.

The nephrectomy specimen was largely replaced by a 6 × 5 cm grayish-white solid areas and 7 × 6 cm grayish-brown cyst containing hemorrhage and necrosis. The tissue samples were fixed in buffered formalin and embedded in paraffin. Additionally, fifteen tissue blocks were taken, and 4-μm-thick tissue sections were stained with hematoxylin-eosin (HE) for routine microscopy. IHC staining was performed using a Ventana Benchmark XT automated IHC stainer. Positive and negative controls were used during the IHC staining. The antibodies involved are all listed in Table 1.

**TABLE 1:** Immunohistochemistry: Antibody specifications.

NO.	Antibody	Manufacturer	Clone	Dilution
1	CKpan	Abcarta	830F6E7	RTU
2	CD10	Abcarta	453F9G7	RTU
3	CyclinD1	Abcarta	332G4S4	RTU
4	RCC	MAIXIN.Bio	PN-15	RTU
5	UroplakinIII	MAIXIN.Bio	SP73	RTU
6	Pax8	Abcarta	282J3C4	RTU
7	CK7	Abcarta	435D4D6	RTU
8	GATA3	Abcarta	255B6B8	RTU
9	CgA	Abcarta	317F1D8	RTU
10	Syn	Abcarta	214A4G5	RTU
11	S100	Abcarta	503F1E9	RTU
12	CD45	Abcarta	154B1H9	RTU
13	P63	Abcarta	281B6A9	RTU
14	AR	MAIXIN.Bio	EP120	RTU
15	β-HCG	MAIXIN.Bio	CG04 + CG05	RTU
16	α-inhibin	MAIXIN.Bio	MX098	RTU
17	SALL4	MAIXIN.Bio	6E3	RTU
18	hPL	MAIXIN.Bio	Polyclonal	RTU
19	PLAP	MAIXIN.Bio	SP15	RTU
20	CDX-2	Abcarta	353G7A8	RTU
21	CK20	Abcarta	120B1A5	RTU
22	PD-L1	Roche	SP263	RTU

RTU, ready-to-use antibody.

**FIGURE 2:**
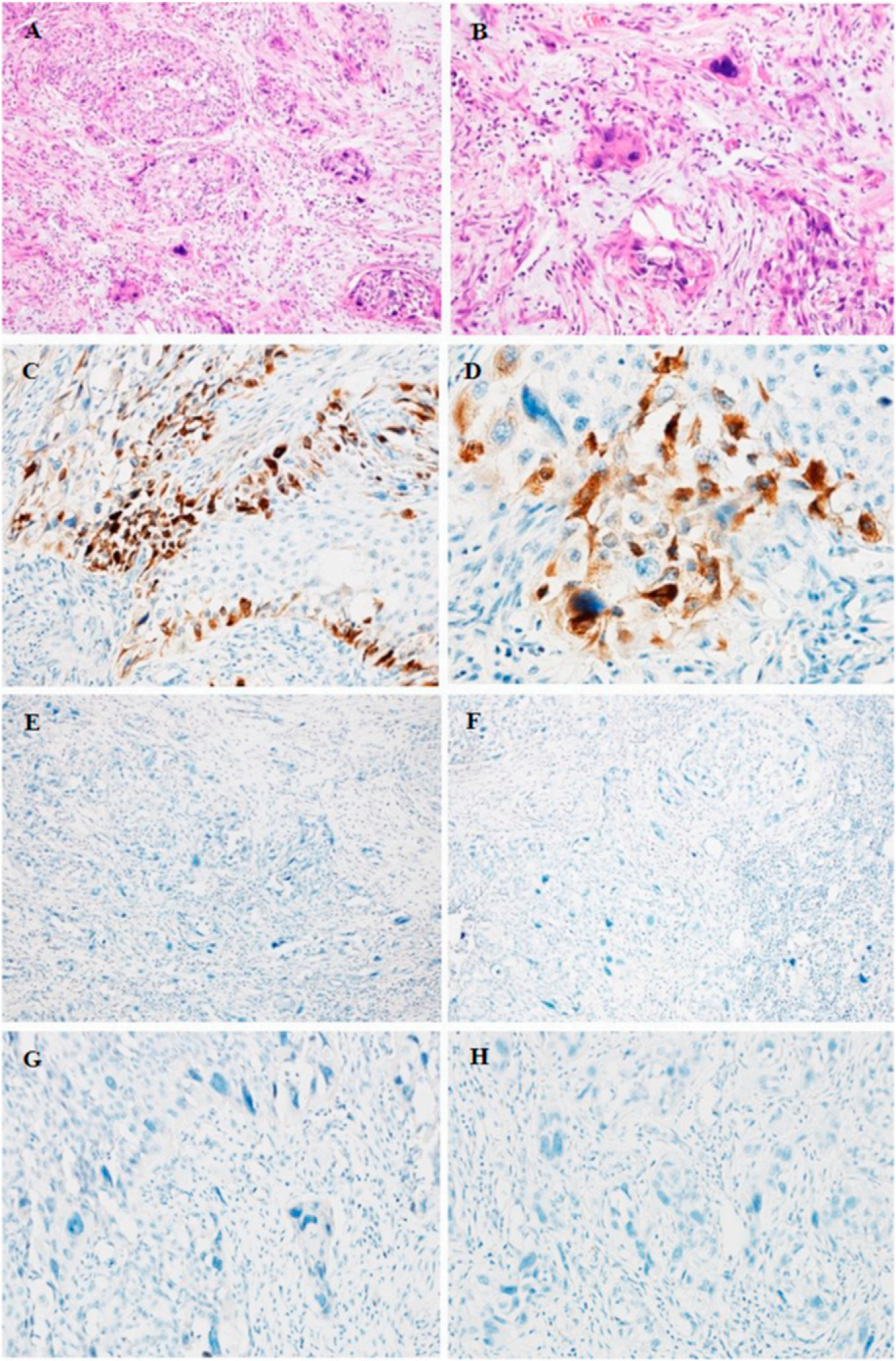
HE **(A, B)** and IHC staining **(C–H)** of UC of the renal pelvis with trophoblastic differentiation. **(A, B)** HE staining showing the areas of UCTD. **(A)** ×200 **(B)** ×400. **(C, D)** Positive staining for β-hCG in syncytiotrophoblastic cells. **(C)** ×200 **(D)** ×400. **(E, F)** Negative staining for PLAP **(E)** and hPL **(F)** in syncytiotrophoblastic cells. **(E, F)** ×100. **(G, H)** Negative staining for CDX2 **(G)** and CK20 **(H)**. **(G, H)** ×200.

Microscopically, a solid HGUC originating from the renal pelvis had numerous scattered bizarre cells or syncytiotrophoblastic cells (Figure 2A). These cells were frequently pleomorphic, with large hyperchromatic nuclei with abundant eosinophilic cytoplasm distributed throughout the UC component (Figure 2B). Hemorrhage, necrosis, and vascular space invasion were prominent features. Tumor cells invaded the renal parenchyma, but not renal capsule. The components of UC were positive for CKpan, CD10 and CyclinD1, but negative for UroplakinIII, RCC, Pax-8, GATA3, Cytokeratin7, AR, CgA, Syn, S-100, CD45, and P63.

The areas of the syncytiotrophoblasts were strongly positive for β-hCG and negative for Placental alkalen phosphatase (PLAP), human placental lactogen (hPL), Sal-like protein 4 (SALL-4), and α-inhibin. In addition, we also performed PD-L1 staining, which showed negative expression of both components. The immunoreaction of β-hCG was localized in the cytoplasm of syncytiotrophoblasts with a diffuse, granular around the periphery of UC nests (Figures 2C, D), while urothelial neoplastic cells were negative. However, syncytiotrophoblasts showed negative for PLAP (Figure 2E) and hPL (Figure 2F). In addition, we also performed CDX2 and CK20 to identify UC with glandular differentiation due to the small amount of pseudo glandular architecture; however, the results of IHC staining were both negative (Figures 2G, H).

**FIGURE 3:**
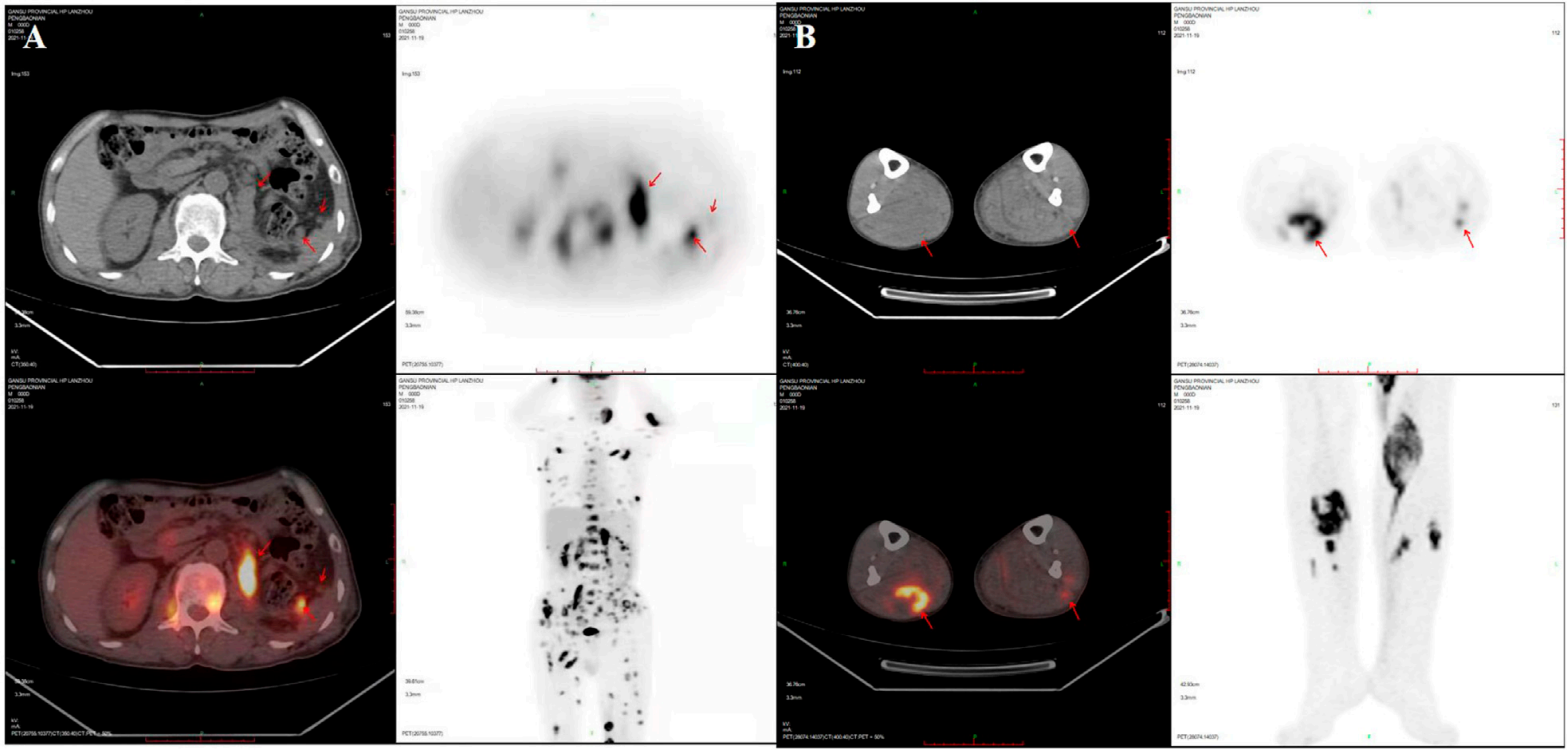
PET-CT scan showed the patient developed multiple nodules of metastasis in the left renal region, extensive systemic muscle, bone, lymph node, liver and bilateral lung metastases. **(A)** Multiple nodules of metastasis in the left renal region, extensive systemic muscle, bone, lymph node, liver and bilateral lung metastases. **(B)** Multiple metastases are seen in bilateral lower limb muscles. SUVmax, maximum of standardized uptake value.

**FIGURE 4:**
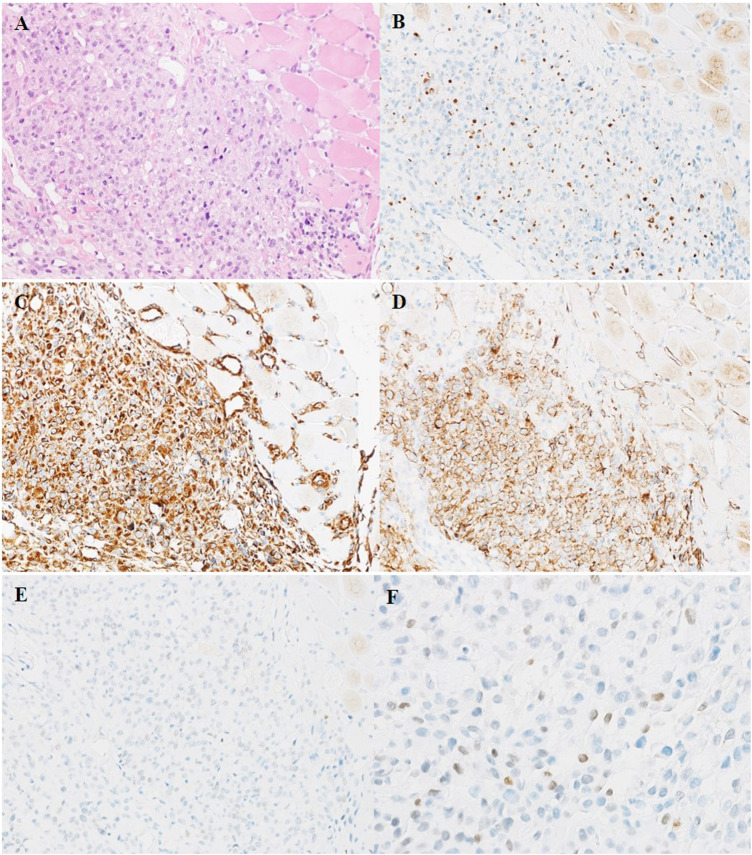
HE **(A)** and IHC staining **(B–F)** of puncture biopsy tissue of muscle metastatic tumor of left lower limb. **(A)** Metastatic UC cells diffusely infiltrate the muscle tissue of left lower limb. **(B)** The nuclei of metastatic tumor cells are scattered positive expression of Ckpan. **(C)** Tumor cells are diffuse strongly positive expression of Vimentin. **(D)** The membrane of metastatic tumor cells are strongly positive expression of CD10. [**(A–D)** ×200]. **(E–F)** The nuclei of metastatic tumor cells are focal positive expression of GATA3. **(E)** ×200 **(F)** ×400.

Three weeks after surgery, postoperative examination of serum β-hCG and tumor markers was carried out. Elevated levels of serum β-hCG of 6.25 U/mL (normal 0–5 U/mL), glycoprotein 125 (CA125) of 76.6 U/mL (normal 0–35 U/mL) and neuron specific enolase (NSE) of 40.39 ng/mL (normal 0–17.0 ng/mL) were found. Serum α-fetoprotein (AFP) was negative. PET-CT scan showed the patient presented multiple nodules of metastasis in the left renal region (SUVmax7.24), extensive systemic muscle (SUVmax14.28), bone (SUVmax7.25), lymph node (SUVmax13.50), liver (SUVmax1.72) and bilateral lung (SUVmax1.28) (Figures 3A, B).A puncture biopsy of a space occupying lesion in lower limb muscle has confirmed metastatic UC (Figures 4A–F). In view of the poor general condition of the patient, bladder perfusion chemotherapy and gemcitabine combined with cisplatin were administered post-surgery.

## Discussion

UCTD, accompanied by production of β-hCG, is a very rare subtype of UC (8). Only seven cases have been reported in English literature to date (Table 2). Similar cases documented in the bladder showed the diagnostic morphologic characteristics of choriocarcinoma, the tumor cells resemble syncytiotrophoblasts and strongly express β-hCG. Extensive necrosis and hemorrhage were also predominant features.

**TABLE 2:** Summary of studies reporting UC of the renal pelvis with trophoblastic differentiation since 1989.

First Author/Year	Age/Sex	Size (cm)	pT	TNM	LVI	Surgical treatment	Evolution (month)
Elias campo/1989	54/M	NG	NG	NG	NG	RN	A6
Winfried Vahlensieck/1991	56/F	3	pT3	pT3pN0M0	NG	NU	A22
Dino Grammatico/1993	84/M	1.5 × 1.0	NG	NG	NG	NU	ND
Andreas Zettl/2002	60/M	15 × 9 × 10	pT4	pT4pNxM1	NG	RN	D1.5
Ovidiu Preda/2012	47/M	6 × 4.7	pT4	pT4pNxM1	NG	NU	A18
Pavlos Msaouel/2017	48/M	9.5	NG	NG	NG	NU	ND
Inês Rolim/2020	82/M	4.5	pT4	NG	NG	RN	A12
Zhuo Wang/2022^a^	55/M	14.3 × 9.4 × 6.8	pT3	pT3pNxM1	(+)	RN	D1

RN, radical nephrectomy; NU, nephroureterectomy; NG, not given; ND, none done; A, alive; D, dead.

^a^ Present report.

In recent years, GATA3 has been recognized as a useful urothelial marker specifically in diagnostic surgical pathology practice (9). Due to the negative expression in this case, we consulted the literature to explain this phenomenon. Firstly, the expression of GATA3 was downregulated in UC, compared with non-neoplastic urothelial tissues(10). In addition, Miyamoto et al. discovered that loss of GATA3 was associated with high-grade and/or muscle-invasive tumors (11). In other words, it was decreased in HGUC and/or muscle-invasive neoplasms. Thirdly, the expression of GATA3 was related to different tumor sites. Inoue et al. found for the first time that the positivity rate of GATA3 was significantly lower in renal pelvic tumor tissues than in ureteral tumor tissues (9). Accordingly, the negativity of GATA3, a promising urothelial marker, does not readily exclude the possibility of UC. Moreover, the expression of GATA3 needs to be further studied by accumulating cases of UCTD.

UCTD represents a rare histological subtype of carcinomas associated with a poor prognosis (12). A recent comparative study of UC and UCTD found a higher risk of recurrence, progression, and death of patients with UCTD of the bladder (12). In our study, there were multiple nodules of metastasis in the left renal region, extensive systemic muscle, bone, lymph node, liver and bilateral lung metastases 3 weeks after surgery. It is suggested that the tumor progresses rapidly and the prognosis may be poor. And this is basically consistent with literature reports.

Reviewing the literature, we found UCTD patients range in age from 47 to 84 years (mean, 61 years), and nearly 75% of patients are older than 50 years in all these cases. Seven out of eight cases occurred in men, including this one (Table 2). Except for the typical morphological features, the IHC of trophoblastic differentiation areas are all strong expression of β-hCG, among which three out of eight expressed α-inhibin, two out of seven expressed hPL, one out of seven expressed SALL4 (Table 3). In our case, the area of syncytiotrophoblasts were strongly expressed β-hCG, but negative for hPL, α-inhibin, PLAP and SALL4 (Table 3). The two components of UC and UCTD represent the heterogeneity of the tumor. In addition, among these cases, the postoperative serum β-hCG was extremely elevated in four cases, and in other cases was not detected. Since the preoperative serum β-hCG was not a routine examination item, it was not measured preoperatively. In our patient, the serum β-hCG was higher than the normal range 3 weeks after surgery (Table 3). Moreover, some studies had confirmed that trophoblastic differentiation is derived from UC. The appearance of morphological change may be due to the gradual emergence of poorly differentiated tumor cells containing placental proteins (3). Earlier hypotheses had been proposed for the histogenesis of UCTD. Some authors had supported that the change occurs through primitive rests of totipotential cells during early embryologic development (1, 13).Other authors had argued that it is a result of dedifferentiation or retrodifferentiation of UC, whereas some had favored that it is a metaplastic phenomenon (3).To date, the well accepted hypothesis of origin is the latter one (14, 15).

**TABLE 3:** Markers of UC of the renal pelvis with trophoblastic differentiation: Review of Literature Since 1989.

Case	Postoperative serum β-HCG (U/mL)	Urothelial carcinoma cells	Syncytiotrophoblastic cells
1	ND	NG	β-HCG (++), hPL (++),SP-1 (+)
2	10.3	NG	β-HCG (+)
3	ND	NG	β-HCG (+)
4	ND	CK7 (+), CK8 (+), CK19 (+), EMA (+), p53 (+), CK20 (−), Uroplakin (−), CEA (−), PLAP (−), β-HCG (−), α-inhibin (−), p21 (−)	CK7 (+), CK8 (+), CK19 (+), EMA (+), p53 (+), β-HCG (+), α-inhibin (+), CK20 (−), Uroplakin (−), CEA (−), PLAP (−), p21 (−)
5	9897.06	CK7(+), p63 (+)	β-HCG (+), hPL (+), α-inhibin (+)
6	5277	AE1/AE3, CK7 (+), GATA-3 (+)	β-HCG (+), SALL4 (+), α-inhibin (+), GATA3 (+), RCC (−), PAX8 (−), P63 (−), CK20 (−), OCT3/4 (−), Glypican-3 (−)
7	ND	NG	β-HCG (+)
8^a^	6.25	CKpan (+), CD10 (+), CyclinD1 (+), AR (−), RCC(−), UroplakinIII (−), Pax8 (−), CK7(−), CgA(−), Syn (−), S100 (−), CD45 (−), P63 (−), GATA3 (−)	β-HCG (+), α-inhibin (−), SALL4 (−), hPL (−), PLAP (−), CDX-2 (−), CK20 (−)

β-HCG, beta-human chorionic gonadotropin; hPL, human placental lactogen; SALL-4, Sal-like protein 4; SP-1, pregnancy-specific beta-1 glycoprotein; NG, not given; ND, not done.

^a^ Present report.

Accordingly, studies have shown that the expression of β-hCG in UC was associated with a poor prognosis (16). Three out of eight patients were classified as pT4 and two as pT3, including this one. Three out of eight patients developed distant metastases, including this one. As in our case, most reported cases were aggressive tumors, with most patients dying shortly after initial diagnosis of widespread metastatic disease. In addition, autopsy studies about UCTD of urinary bladder showed that only tumor cells of trophoblastic differentiation were found in the multiple metastatic sites after gemcitabine and oxaliplatin treatment, and no UCs (17). Chemotherapy regimen basically consistent with literature reports is adopted. During the postoperative follow-up, the patient passed away just 33 days after surgery.

## Conclusion

In conclusion, we described UC of the renal pelvis with trophoblastic differentiation, which is an extremely rare but aggressive malignancy with poor prognosis. The morphologic and IHC findings of this report and the literature review suggest that the trophoblastic differentiation evolves from UC. This type of carcinoma is characterized by syncytiotrophoblastic cells and abnormal strongly expressed with β-hCG. Due to the limited number of cases reported in the English-language literature to date, it is of great significance for further study of the treatment and prognosis of the patients to give a definite diagnosis according to their morphological and IHC staining characteristics.

## Data Availability

The original contributions presented in the study are included in the article/supplementary material, further inquiries can be directed to the corresponding author.
